# Synergistic effects of probiotics and low-FODMAP diet on clinical and inflammatory outcomes in ulcerative colitis: a retrospective cohort study

**DOI:** 10.3389/fmed.2026.1801783

**Published:** 2026-05-28

**Authors:** Shan Jia, XiaoLong Yu, BaoSheng Bao, BinBin Liu

**Affiliations:** 1 Twelve Wards of Zhejiang Hospital Sandun Campus, Zhejiang, China; 2 Department of Nutrition, Zhejiang Hospital, Zhejiang, China; 3 Department of Rheumatology and Immunology, Zhejiang Hospital, Zhejiang, China

**Keywords:** clinical outcomes, gut-microbiota-immune axis, Inflammation, Low-FODMAP diet, Probiotics, ulcerative colitis

## Abstract

**Background:**

Ulcerative colitis (UC) is a chronic inflammatory bowel disease characterized by mucosal inflammation, in which gut dysbiosis and immune imbalance play pivotal roles. Dietary and microbiota-targeted interventions, particularly the low-FODMAP (fermentable oligosaccharides, disaccharides, monosaccharides, and polyols) diet and probiotics, have individually shown benefits in symptom control and inflammation modulation. However, evidence on their combined impact remains limited.

**Methods:**

This retrospective cohort study included 80 adults with mild-to-moderate UC managed at a tertiary inflammatory bowel disease center between 2022 and 2025. Patients were stratified into four exposure groups: low-FODMAP + probiotic, low-FODMAP only, probiotic only, and standard care. Clinical and biochemical outcomes were assessed over approximately 8 weeks. Primary endpoints included change in Mayo score and clinical remission. Secondary outcomes included changes in C-reactive protein (CRP) and fecal calprotectin (FCal).

**Results:**

The combined low-FODMAP + probiotic group showed the greatest reduction in Mayo score (−2.4 ± 0.8; *p* < 0.001) and CRP (−3.5 ± 1.8 mg/L), with 60% achieving clinical remission. A significant improvement in FCal (−180 ± 70 μg/g) was also observed in this group.

**Conclusion:**

A combined low-FODMAP diet and probiotic regimen was associated with the most favorable clinical and inflammatory outcomes in this cohort of adults with mild-to-moderate UC. Because a formal statistical interaction test was not performed, these findings should not be interpreted as proof of synergy, but rather as evidence of an observed combined benefit in this retrospective study. This integrated nutritional–microbial strategy represents a promising adjunctive approach for personalized UC management.

## 1 Introduction

Ulcerative colitis (UC) is a chronic, relapsing condition characterized by inflammation of the colon's mucosal lining. It is widely understood that the pathogenesis of UC results from an intricate interplay of genetic predisposition, environmental influences, dysbiosis of the gut microbiota, and immune system dysfunction. Although significant progress has been made in pharmacologic treatment, including the use of aminosalicylates, corticosteroids, immunomodulators, and biologic agents, a considerable number of patients still experience incomplete remission or suffer from adverse effects. This highlights the ongoing need for effective and complementary therapeutic strategies (1).

In recent years, there has been growing interest in dietary interventions and microbiota-targeted therapies as potential strategies for managing UC. Among these approaches, the low-FODMAP diet (which restricts fermentable oligosaccharides, disaccharides, monosaccharides, and polyols) has demonstrated effectiveness in alleviating gastrointestinal symptoms in individuals with both inflammatory bowel disease (IBD) and irritable bowel syndrome (IBS). The diet works by limiting the intake of poorly absorbed, fermentable carbohydrates that are rapidly metabolized by gut bacteria, thereby reducing the production of gas, intestinal distension, and associated gastrointestinal symptoms (2). Randomized controlled trials have shown that the low-FODMAP diet effectively alleviates gastrointestinal symptoms and enhances quality of life in patients with quiescent IBD or IBS (3, 4). However, emerging evidence suggests that prolonged adherence to the low-FODMAP diet may reduce the abundance of beneficial commensal bacteria, such as Bifidobacterium and Faecalibacterium prausnitzii, potentially disrupting the gut microbiota and impairing long-term mucosal immune homeostasis (5).

Probiotics, defined as live microorganisms that confer health benefits when administered in sufficient quantities, represent a promising therapeutic approach for UC. Numerous studies have shown that probiotics can restore microbial balance, improve epithelial barrier integrity, and reduce mucosal inflammation (6). Preclinical models have demonstrated that supplementation with Lactobacillus plantarum alleviates colitis by modulating inflammatory cytokine levels, enhancing tight junction protein expression, and promoting the production of short-chain fatty acids (SCFAs) (7). Clinical trials and systematic reviews have further substantiated that probiotic therapy has the potential to reduce disease activity and sustain remission in patients with (UC). However, the outcomes are influenced by factors such as probiotic strain, dosage, and the stage of the disease (1).

Considering that the low-FODMAP diet reduces both luminal fermentation and the population of beneficial microbes, while probiotics help replenish these beneficial species and support immune regulation, a combined approach could potentially offer synergistic therapeutic effects. Evidence from IBS populations indicates that the co-administration of probiotics with a low-FODMAP diet may help mitigate the reduction in beneficial bacteria caused by dietary restrictions, while also sustaining symptom relief (8, 9). However, studies specifically examining this combined intervention in UC are limited. Most of the current research has been focused on IBS or preclinical models, highlighting a significant gap in knowledge regarding the real-world impact of low-FODMAP dietary counseling and probiotic supplementation in UC patients.

Furthermore, although both interventions are commonly recommended in clinical practice, their impact on inflammatory biomarkers, mucosal healing, and gut microbial metabolites especially SCFAs is not well understood. The complex interactions between diet, microbiota composition, and immune modulation in UC remain inadequately defined (6, 10). A clearer understanding of these relationships is crucial for optimizing microbiota-targeted interventions and developing personalized therapeutic strategies aimed at modulating the gut microbiota–immune axis.

Therefore, this retrospective cohort study aims to evaluate whether exposure to probiotics and/or a low-FODMAP diet is associated with improved clinical and inflammatory outcomes in adults with mild-to-moderate UC.

## 2 Methods

### 2.1 Study design and setting

This was a retrospective, longitudinal, observational cohort study conducted at a tertiary inflammatory bowel disease (IBD) referral center. The study utilized routinely collected clinical data from gastroenterology and dietetic clinics between January 2022 and February 2025. The study protocol was approved by the Institutional Review Board, and a waiver of informed consent was obtained where applicable, in accordance with national ethical regulations. All patient data were de-identified before analysis.

### 2.2 Study population

Eligible participants for this study were adults between the ages of 18 and 70 who had a confirmed diagnosis of ulcerative colitis (UC), established through clinical, endoscopic, and histopathological evaluations. To be included, patients needed to have mild-to-moderate disease activity at baseline and must have been on stable maintenance therapy for at least 8 weeks prior to enrollment. Additionally, each participant had to have had at least one documented treatment episode of approximately 8 weeks, with corresponding follow-up data available, during which exposure to a low-FODMAP diet and/or probiotic supplementation was clearly recorded. The inclusion criteria required participants to be aged 18 to 70 years, have a confirmed diagnosis of UC, and exhibit mild-to-moderate disease activity at baseline, with documented dietary or probiotic exposure and both baseline and follow-up disease activity data available.

Exclusion criteria included those with severe or fulminant UC that required hospitalization or surgery at baseline, as well as individuals who underwent major therapeutic changes, such as the initiation of biologic therapy or colectomy, during the 8-week window of the study. Participants with incomplete records, lacking reliable clinical or follow-up data, were also excluded. Additionally, any patients with concomitant gastrointestinal diseases, such as active infections, that could potentially confound the study outcomes were excluded from participation.

### 2.3 Exposure assessment

Patients were categorized into four distinct exposure groups based on documented dietary counseling and probiotic use, as recorded in the electronic medical records (EMR). The groups included: 1) Low-FODMAP diet combined with probiotics, 2) Low-FODMAP diet only (without probiotics), 3) Probiotics only (with a standard diet), and 4) Neither low-FODMAP nor probiotic (control group).

### 2.4 Low-FODMAP diet exposure

Low-FODMAP diet exposure was defined as documented, structured dietary counseling provided by a gastroenterologist or dietitian, and at least one subsequent follow-up encounter recording patient-reported full or partial adherence to the prescribed regimen. As this retrospective analysis relied on routinely collected clinical documentation, validated adherence tools such as food frequency questionnaires or prospective 3-day food diaries were not uniformly available. Therefore, dietary exposure classification was based on pragmatic chart documentation and should not be interpreted as a standardized quantitative measure of low-FODMAP compliance.

### 2.5 Probiotic exposure

Probiotic exposure was defined by the prescription or recommendation of Pei Fei Kang or or other multi-strain probiotic products noted in the electronic medical record, including formulations containing organisms such as *Bifidobacterium, Lactobacillus*, and *Enterococcus*. Because this was a retrospective study, the exact strain composition, formulation equivalence, and CFU dose were not consistently available for all participants and therefore could not be standardized analytically. The recorded duration of probiotic use was approximately 8 weeks. Thus, the probiotic group should be interpreted as representing heterogeneous real-world probiotic exposure rather than a uniform strain-specific intervention.

### 2.6 Data sources and collection

Clinical data were extracted from several sources, including the electronic medical records and IBD clinic notes, as well as documentation from dietitians and dietary assessment forms. Laboratory data were retrieved from routine clinical records, including biomarkers such as C-reactive protein (CRP) and fecal calprotectin. Disease activity scoring and histological data were obtained from endoscopy and pathology reports available in the electronic medical records. No additional biobank samples were accessed or analyzed for this retrospective study.

Data were systematically collected using a standardized electronic data extraction form and were subsequently entered into a secure REDCap database, which included built-in quality control measures, range checks, and audit trails to ensure data accuracy and integrity.

### 2.7 Outcome measures

The primary outcome of the study was the change in either total or partial Mayo score from baseline (T0) to approximately 8 weeks (T1). This measure was used to assess the clinical severity of ulcerative colitis and its response to the interventions being studied.

Secondary outcomes included the proportion of patients achieving clinical remission or response at T1, as well as changes in biomarkers such as C-reactive protein (CRP) and fecal calprotectin between baseline and follow-up. Additionally, the study assessed changes in gastrointestinal symptom burden, focusing on measures such as stool frequency, urgency, and abdominal pain. The need for treatment escalation, such as the initiation of corticosteroids or hospitalization, during the follow-up period was also considered an important secondary outcome.

### 2.8 Exposure and outcome windows

The baseline (T0) visit was defined as the initial visit during which exposure to the low-FODMAP diet and/or probiotic supplementation was first documented, or a corresponding visit for standard-care control participants. Follow-up (T1) was defined as the closest clinical evaluation conducted approximately 8 ± 2 weeks after T0, provided that sufficient clinical and biomarker data were available. Because follow-up data beyond approximately 8 weeks were not sufficiently complete or standardized across patients, the present analysis was restricted to the T0 to T1 interval and did not include a formal evaluation of 3- to 6-month outcomes.

### 2.9 Statistical analysis

Continuous variables were presented as mean ± standard deviation (SD) or median (interquartile range), as appropriate according to data distribution, while categorical variables were expressed as counts and percentages.

The primary analysis compared changes in Mayo score across the exposure groups using analysis of covariance (ANCOVA) or multivariable linear regression. These models were adjusted for baseline Mayo score, age, sex, disease duration, and key medications to account for measured confounders. Because treatment allocation was determined by routine clinical practice rather than randomization, the multivariable models were additionally intended to partially mitigate confounding by indication and selection bias through adjustment for baseline disease activity and other major measured clinical covariates. However, potentially relevant factors such as patient motivation, health literacy, adherence behavior, and intensity of dietary follow-up were not consistently available in the retrospective record and therefore could not be directly controlled.

For secondary analyses, logistic regression was used to estimate odds ratios for achieving clinical remission or requiring treatment escalation. Changes in biomarkers, including C-reactive protein (CRP) and fecal calprotectin, between baseline and follow-up were analyzed using paired *t*-tests or appropriate nonparametric tests, depending on data distribution.

Missing data were handled using multiple imputation where appropriate. Sensitivity analyses were performed to compare results from imputed datasets with complete-case analyses.

All statistical analyses were performed using R software (version 4.3.0) or SPSS (version 29). A two-sided *p*-value < 0.05 was considered statistically significant.

### 2.10 Data management and quality assurance

All extracted data were de-identified and stored in a secure, password-protected REDCap system. Quality control included double-data entry for 10% of records and logic/range validation for key variables. Discrepancies were resolved by consensus between two independent reviewers.

### 2.11 Ethical considerations

This retrospective study involved no intervention or additional patient risk. Ethical approval was obtained from the institutional review board prior to data collection. All analyses used anonymized data, and findings will be reported in aggregate form to ensure confidentiality.

## 3 Results

### 3.1 Study population

A total of 80 adult patients with mild-to-moderate ulcerative colitis (UC) were included in this retrospective cohort analysis. Participants were stratified into four groups based on documented exposure to a low-FODMAP diet and/or probiotic supplementation: low-FODMAP + probiotic (*n* = 20), low-FODMAP only (*n* = 20), probiotic only (*n* = 20), and standard care/control (*n* = 20). The mean age of participants was 42 ± 10 years, with a balanced sex distribution (52% male, 48% female). Baseline demographic and clinical characteristics were comparable across the groups, with no significant differences observed in disease duration, extent of colitis, or concomitant medications (Table 1).

**Table 1:** Baseline demographic and clinical characteristics of study participants.

Variable	Low-FODMAP + probiotic (*n* = 20)	Low-FODMAP only (*n* = 20)	Probiotic only (*n* = 20)	Control (*n* = 20)
Number of patients (*n*)	20	20	20	20
Age (years, mean ± SD)	42 ± 9	41 ± 10	43 ± 11	42 ± 10
Male sex (%)	55	50	50	55
Baseline Mayo score (mean ± SD)	5.6 ± 1.0	5.5 ± 1.1	5.4 ± 0.9	5.5 ± 1.0
Follow-up Mayo score (mean ± SD)	3.2 ± 1.1	3.8 ± 1.2	3.5 ± 1.0	4.5 ± 1.1
Δ Mayo (mean ± SD)	−2.4 ± 0.8	−1.7 ± 1.0	−1.9 ± 0.9	−1.0 ± 0.9
Baseline CRP (mg/L, mean ± SD)	8.2 ± 2.9	7.9 ± 3.0	8.0 ± 3.1	7.8 ± 2.8
Follow-up CRP (mg/L, mean ± SD)	4.7 ± 1.8	5.9 ± 2.0	5.5 ± 1.9	6.6 ± 2.0
Δ CRP (mg/L, mean ± SD)	−3.5 ± 1.8	−2.0 ± 1.7	−2.5 ± 1.5	−1.2 ± 1.3
Baseline Fecal Calprotectin (μg/g, mean ± SD)	410 ± 95	395 ± 110	405 ± 100	400 ± 105
Follow-up Fecal Calprotectin (μg/g, mean ± SD)	230 ± 70	265 ± 80	255 ± 75	310 ± 85
Δ Fecal Calprotectin (μg/g, mean ± SD)	−180 ± 70	−130 ± 75	−150 ± 60	−90 ± 65

### 3.2 Clinical outcomes

#### 3.2.1 Change in disease activity

**Figure 1:**
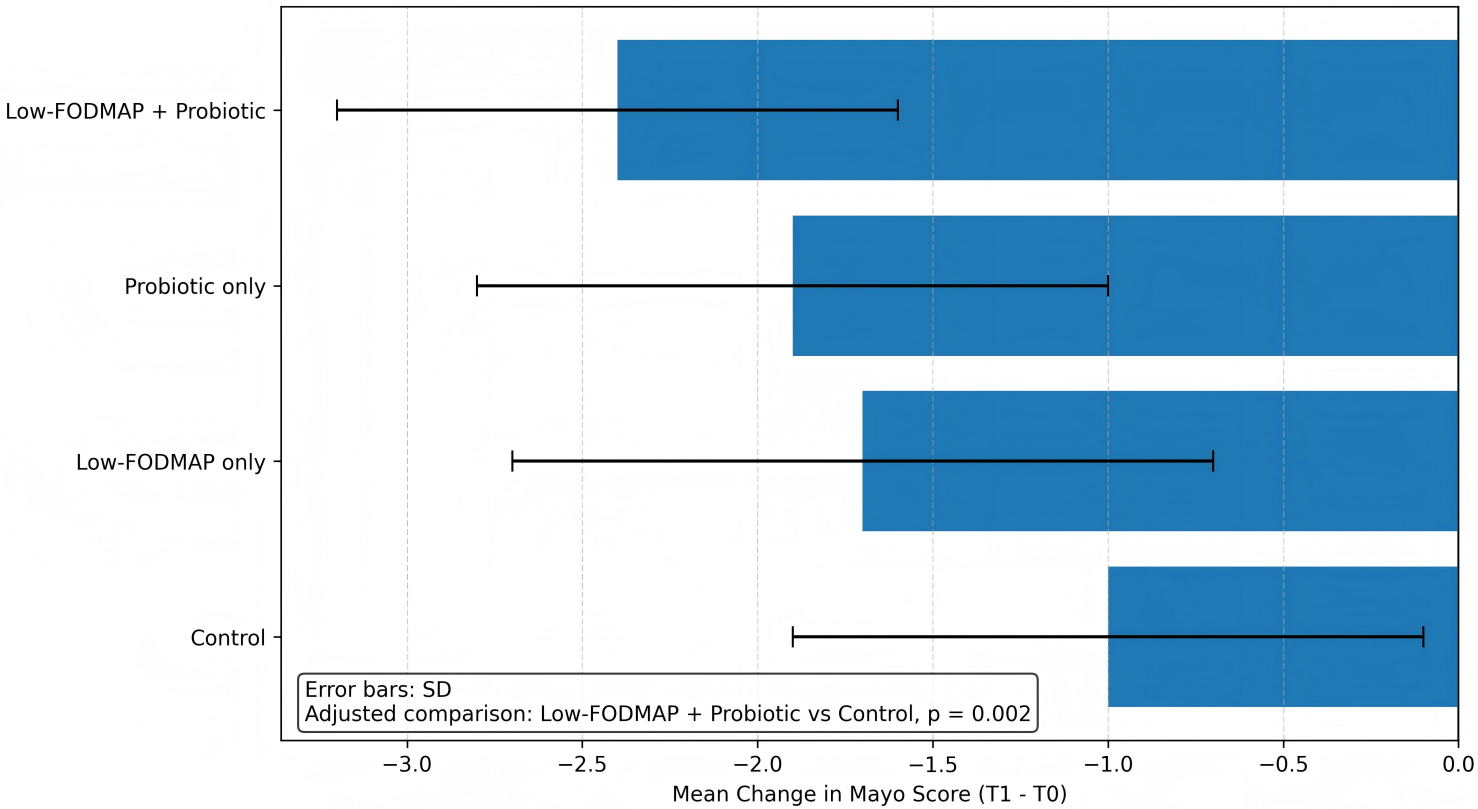
Change in clinical disease activity by exposure group. Mean change in Mayo score from baseline to follow-up among patients with mild-to-moderate ulcerative colitis stratified by exposure group: low-FODMAP + probiotic, probiotic only, low-FODMAP only, and standard care/control. Bars represent group mean changes, and error bars indicate standard deviations. More negative values indicate greater improvement in clinical disease activity.

Across the entire cohort, a significant improvement in clinical disease activity was observed after 8 weeks. The mean Mayo score decreased from 5.5 ± 1.0 at baseline to 3.5 ± 1.2 at follow-up (*p* < 0.001). The low-FODMAP + probiotic group exhibited the largest mean reduction in Mayo score (−2.4 ± 0.8), followed by the probiotic-only group (−1.9 ± 0.9) and the low-FODMAP-only group (−1.7 ± 1.0), while the control group showed a more modest improvement (−1.0 ± 0.9) (Figure 1).

After adjusting for age, sex, baseline disease activity, and maintenance therapy, the low-FODMAP + probiotic group demonstrated a statistically significant greater reduction in Mayo score compared with the control group (adjusted β = −1.3, 95% CI: −2.0 to −0.6, *p* = 0.002).

### 3.3 Clinical remission and response

Clinical remission, defined as a partial Mayo score ≤ 2 without rectal bleeding, was achieved in 60% of patients in the low-FODMAP + probiotic group. In comparison, 40% of patients in the probiotic-only group, 35% in the low-FODMAP-only group, and 25% in the control group reached clinical remission. This suggests that the combination of a low-FODMAP diet and probiotics was more effective in inducing clinical remission than either intervention alone or standard care (Table 2).

**Table 2:** Clinical and inflammatory outcomes at 8 weeks across exposure groups.

Outcome	Low-FODMAP + probiotic	Probiotic only	Low-FODMAP only	Control	*p*-value
Δ Mayo score (mean ± SD)	−2.4 ± 0.8	−1.9 ± 0.9	−1.7 ± 1.0	−1.0 ± 0.9	0.01
Clinical remission (%)	60	40	35	25	0.03
Δ CRP (mg/L, mean ± SD)	−3.5 ± 1.8	−2.5 ± 1.5	−2.0 ± 1.7	−1.2 ± 1.3	0.02
Δ Fecal Calprotectin (μg/g, mean ± SD)	−180 ± 70	−150 ± 60	−130 ± 75	−90 ± 65	0.04
Treatment escalation (%)	5	10	15	20	0.08

In addition to clinical remission, the low-FODMAP + probiotic group also demonstrated the highest clinical response rate, defined as a ≥30% reduction in Mayo score, at 85%, compared to 55% in the control group. This substantial difference was statistically significant (*p* = 0.01), highlighting the potential synergistic effect of the low-FODMAP diet and probiotics in improving disease activity. These findings suggest that the combined intervention may provide a more effective therapeutic strategy for managing mild-to-moderate ulcerative colitis compared to probiotics or a low-FODMAP diet alone (Table 2).

### 3.4 Inflammatory and biomarker outcomes

#### 3.4.1 C-Reactive Protein (CRP)

**Figure 2:**
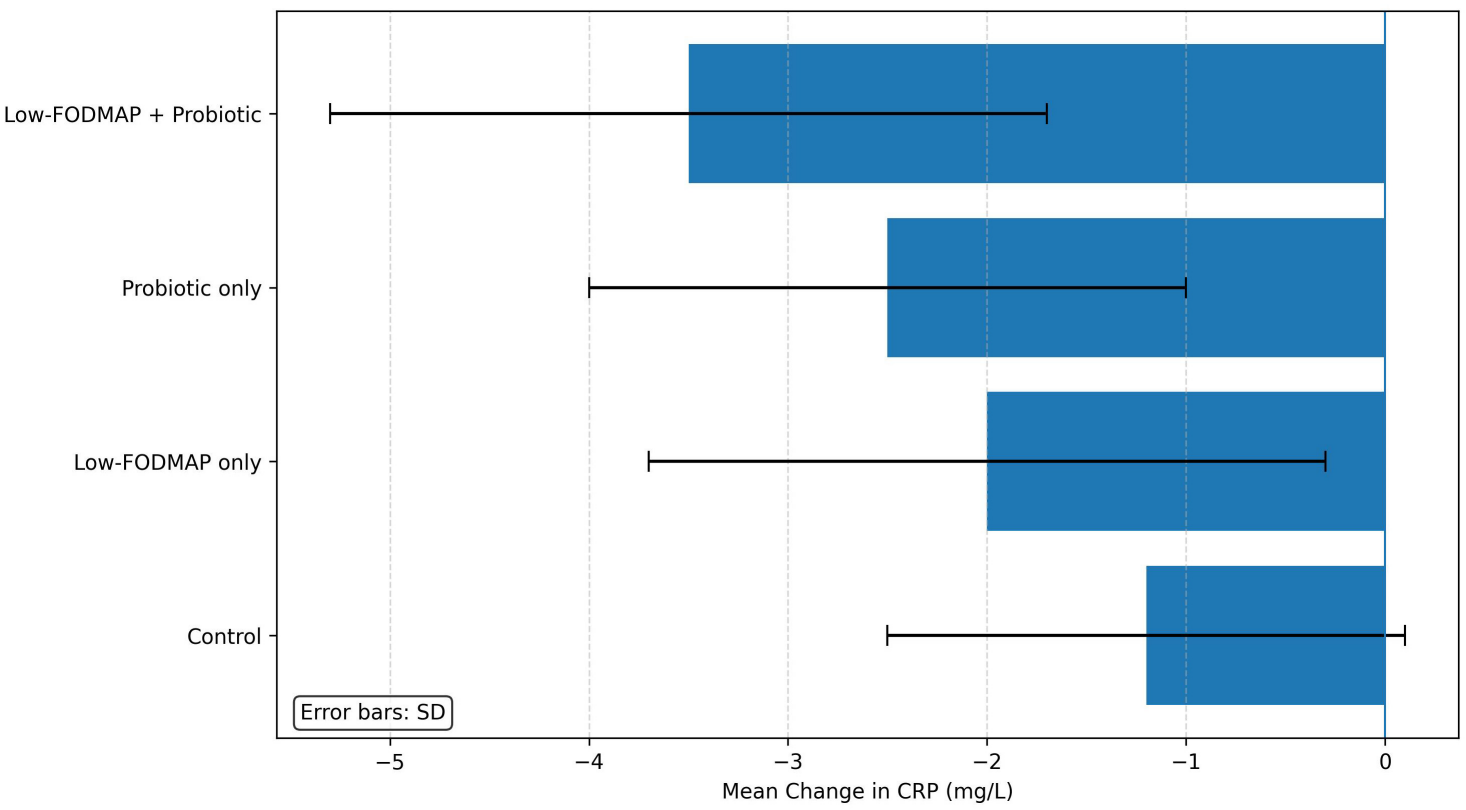
Change in systemic inflammation by exposure group. Mean change in C-reactive protein (CRP) from baseline (T0) to approximately 8 weeks (T1) among patients with mild-to-moderate ulcerative colitis stratified by exposure group: low-FODMAP + probiotic, probiotic only, low-FODMAP only, and standard care/control. Bars represent group mean changes, and error bars indicate standard deviations (SD). More negative values indicate greater reduction in systemic inflammation. The low-FODMAP + probiotic group showed the largest mean reduction in CRP among the four groups.

Mean CRP levels decreased significantly from 8.0 ± 3.0 mg/L at baseline to 5.0 ± 2.0 mg/L at follow-up across all patients (*p* < 0.001) (Table 1). The most significant reduction in CRP occurred in the low-FODMAP + probiotic group (−3.5 ± 1.8 mg/L), followed by the probiotic-only group (−2.5 ± 1.5 mg/L) and the low-FODMAP-only group (−2.0 ± 1.7 mg/L). The control group exhibited a modest improvement (−1.2 ± 1.3 mg/L) (Figure 2). These findings suggest that the combined low-FODMAP and probiotic intervention was most effective in reducing systemic inflammation, as indicated by CRP levels.

#### 3.4.2 Fecal calprotectin (FCal)

Fecal calprotectin levels decreased significantly from a mean of 400 ± 100 μg/g at baseline to 250 ± 80 μg/g at follow-up (*p* < 0.001) across the entire cohort (Table 1). The most pronounced reduction in fecal calprotectin was observed in the low-FODMAP + probiotic group (−180 ± 70 μg/g), followed by the probiotic-only group (−150 ± 60 μg/g), the low-FODMAP-only group (−130 ± 75 μg/g), and the control group (−90 ± 65 μg/g). These results suggest that the combination of a low-FODMAP diet and probiotics was the most effective intervention in reducing fecal calprotectin levels, a key biomarker of intestinal inflammation.

Correlation analyses revealed that changes in fecal calprotectin (FCal) were significantly associated with reductions in Mayo score (*r* = 0.68, *p* < 0.001) and C-reactive protein (CRP) (*r* = 0.51, *p* < 0.01) (Table 1, Figure 2). These findings suggest a strong association between improvements in gut inflammation (measured by FCal), clinical disease activity (as measured by the Mayo score), and systemic inflammation (as measured by CRP).

### 3.5 Adverse events and safety

No adverse events related to probiotic or dietary interventions were documented in the retrospective record review. No patients required hospitalization or colectomy during the 8-week observation period. Minor self-limited gastrointestinal discomfort was reported in three patients (two in the probiotic-only group and one in the low-FODMAP group). Because safety data were derived from routine electronic medical record documentation rather than prospective adverse-event monitoring, mild or transient symptoms may have been under-reported.

## 4 Discussion

This retrospective cohort study found that documented exposure to a combined low-FODMAP diet and probiotic regimen was associated with greater improvement in clinical disease activity and inflammatory markers (CRP and fecal calprotectin) than either intervention alone or standard care in patients with mild-to-moderate ulcerative colitis (UC). These findings add to growing evidence supporting integrated dietary and microbial interventions as a complementary therapeutic approach in UC management.

### 4.1 Integration with existing literature

Dietary interventions play a critical role in modulating intestinal inflammation and microbiota composition in inflammatory bowel disease (IBD) (11). The low-FODMAP diet, designed to reduce fermentable carbohydrate intake, has been shown to alleviate gastrointestinal symptoms and improve quality of life in UC and Crohn's disease (12). In a pivotal randomized controlled trial, Cox et al. (3) found that 52% of patients with quiescent IBD on a low-FODMAP diet achieved adequate symptom relief compared with 16% on a control diet, with notable reductions in abdominal pain and bloating (3). However, this diet also decreased beneficial bacterial species such as *Bifidobacterium adolescentis* and *Faecalibacterium prausnitzii*, underscoring potential microbiota depletion as a limitation of long-term restriction (3, 5).

Our study addresses this gap by examining probiotic supplementation in routine clinical practice, although the heterogeneity of formulations in this cohort limits strain-specific mechanistic interpretation. Milajerdi et al. reported similar benefits of a low-FODMAP regimen, including reduced fecal calprotectin and serum TNF-α, accompanied by an increased abundance of *F. prausnitzii* (2). These findings, in concert with our results, suggest that dietary modulation can attenuate mucosal inflammation through microbial mechanisms, but the addition of probiotics may preserve beneficial species and further amplify the anti-inflammatory effect.

Probiotic therapy has shown increasing promise as an adjunctive strategy for UC, although strain-specific efficacy may vary substantially across formulations. Wu et al. (7) demonstrated that *Lactobacillus plantarum* HNU082 improved epithelial integrity and reduced proinflammatory cytokines (IL-6, TNF-α) while increasing IL-10 and short-chain fatty acid (SCFA) levels in murine UC models. Similarly, Huang et al. summarized evidence that probiotic strains such as *Bifidobacterium infantis* and *Lactobacillus rhamnosus* modulate the NF-κB and JAK/STAT pathways, reducing inflammatory signaling in UC (13). Our observed reduction in CRP is consistent with these mechanistic pathways, reinforcing that probiotics improve immune regulation and gut barrier function.

In the current study, the combination group showed the most favorable outcomes in this cohort, although the possibility that this pattern was influenced in part by selection bias, healthy user bias, or residual confounding cannot be excluded, an observation echoed in other disorders of the gut–brain and gut–immune axes. Liu et al. (9) found that co-administration of probiotics with a low-FODMAP diet significantly improved microbial diversity, SCFA production, and symptom severity in IBS patients compared to either treatment alone. This parallel supports the hypothesis that diet and probiotics interact additively to modulate the microbiota and inflammatory milieu.

Moreover, recent reviews highlight that FODMAP restriction, while symptomatically effective, may worsen dysbiosis when used in isolation (9). Simoes et al. (12) cautioned that long-term adherence to low-FODMAP regimens can lead to reduced microbial diversity and potential micronutrient deficiencies.

Beyond symptom improvement, microbiota-targeted therapy is increasingly recognized as a disease-modifying approach in UC (14, 15). Puca et al. (10) reviewed evidence that both low-FODMAP and Mediterranean diets can reduce inflammation and restore microbial equilibrium in chronic pouchitis and UC. Similarly, Bilal et al. (16) emphasized that probiotics and synbiotics can regulate host immune homeostasis by downregulating NF-κB and MAPK pathways, thereby preventing epithelial injury.

Finally, the emerging synbiotic paradigm, combining probiotics with prebiotic or diet-based substrates, has been proposed as a next-generation UC therapy. Ashique et al. (17) noted that synbiotics can reduce reactive oxygen species (ROS) and boost antioxidant enzymes, offering additional cytoprotective effects in UC. The dual dietary–probiotic strategy in our study parallels this model, suggesting that the low-FODMAP diet may serve as a compatible substrate for probiotic metabolic activity, collectively enhancing anti-inflammatory and mucosal healing effects.

### 4.2 Mechanistic interpretation

While this study did not perform direct microbiota or metabolomic analyses, the observed clinical and biomarker improvements are consistent with the hypothesized microbiota–immune axis modulation reported in prior literature (6, 7, 9, 10). Thus, the combined exposure group showed the most favorable clinical outcomes in this cohort; however, a formal statistical test for interaction between diet and probiotic exposure was not performed, and therefore the results should not be interpreted as proof of synergy. The observed associations should therefore be interpreted in the context of pragmatic real-world exposure definitions, particularly because dietary adherence could not be quantified using standardized instruments. In addition, because follow-up data beyond approximately 8 weeks were not sufficiently complete or standardized for formal analysis, the present study is limited to short-term outcomes and cannot determine whether these observed benefits were durable over 3 to 6 months.

### 4.3 Clinical implications

From a clinical standpoint, these findings underscore the potential of integrated nutritional–microbial strategies to complement pharmacologic UC therapy. Unlike corticosteroids or biologics, which target immune pathways directly, diet–probiotic combinations act at the interface of microbial metabolism and immune signaling. This may offer a safe, non-pharmacologic adjunct for symptom control, mucosal healing, and possibly prevention of relapse. Furthermore, these interventions may reduce dependency on systemic immunosuppressants, which carry notable side effects.

### 4.4 Limitations

This study has several limitations. First, its retrospective observational design precludes causal inference and introduces the possibility of selection bias and residual confounding. In particular, exposure to a low-FODMAP diet and/or probiotics was determined by clinician recommendation and patient uptake rather than random allocation. As a result, patients receiving the combined intervention may have differed systematically from those in the other groups in ways not fully captured by the medical record, including health literacy, motivation, adherence capacity, engagement with multidisciplinary care, and other “healthy user” characteristics. To reduce this risk, we restricted the cohort to patients with mild-to-moderate UC on stable maintenance therapy, excluded major treatment changes during follow-up, and adjusted analyses for baseline Mayo score, age, sex, disease duration, and concomitant medications. Nevertheless, residual confounding from unmeasured behavioral and care-access factors likely remains. Second, adherence to the low-FODMAP diet was not measured using validated dietary assessment methods such as food frequency questionnaires or prospective food diaries. Reliance on routine follow-up documentation as a proxy for adherence is inherently subjective, may not accurately reflect the degree of dietary compliance, and represents an important limitation of the study. Third, dietary adherence was assessed from routine documentation and may have been imperfectly measured, introducing possible misclassification and recall bias. Fourth, the sample size limited subgroup analyses by disease extent and treatment regimen. Fifth, no direct microbiota, metabolomic, or cytokine profiling was performed, limiting mechanistic interpretation. Fifth, probiotic exposure was heterogeneous across the cohort, and exact strain composition, formulation details, and CFU dosage were not uniformly available in the retrospective record. This limits reproducibility, reduces mechanistic precision, and prevents conclusions about the effects of any single probiotic strain or formulation. Sixth, formal testing for statistical interaction between the low-FODMAP diet and probiotic exposure was not performed; therefore, our findings reflect observed differences across exposure groups and should not be interpreted as proof of synergistic effects. Seventh, although longer-term follow-up would be clinically informative in UC, data beyond the approximately 8-week observation window were not sufficiently complete or standardized for formal analysis. Therefore, the study cannot address the durability of response, sustained remission, or relapse beyond short-term follow-up. Eighth, safety outcomes were assessed through retrospective chart review rather than prospective adverse-event surveillance. As a result, mild or transient side effects, such as bloating, gas, or abdominal discomfort, may have been under-reported unless they were specifically elicited during follow-up or documented in the medical record. Finally, although UC diagnosis was established using standard clinical, endoscopic, and histopathological criteria, histopathology was not uniformly required in every case, which may affect generalizability. Prospective randomized studies with standardized intervention protocols and richer behavioral and biological covariate capture are needed to validate these findings.

## 5 Conclusion

In conclusion, our study suggests that combined exposure to a low-FODMAP diet and probiotic supplementation is associated with greater improvement in clinical and inflammatory outcomes than either intervention alone in adults with mild-to-moderate UC. Given the retrospective design and potential for residual confounding, these findings should be interpreted as hypothesis-generating and require confirmation in prospective randomized studies. Future research should prioritize identifying optimal diet–probiotic combinations, determining patient phenotypes that respond best to these interventions, and evaluating their long-term safety and efficacy through large-scale randomized clinical trials.

## Data Availability

The original contributions presented in the study are included in the article/supplementary material, further inquiries can be directed to the corresponding author.
